# Alpaca (*Vicugna pacos*) Husbandry and Their Welfare

**DOI:** 10.3390/ani15213092

**Published:** 2025-10-24

**Authors:** Renata Pilarczyk, Małgorzata Bąkowska, Bogumiła Pilarczyk, Agnieszka Tomza-Marciniak, Beata Seremak, Jan Udała, Paulius Matusevičius, Ramutė Mišeikienė

**Affiliations:** 1 Laboratory of Biostatistics, Bioinformatics and Animal Research, Faculty of Biotechnology and Animal Husbandry, West Pomeranian University of Technology in Szczecin, Klemensa Janickiego 29, 71-270 Szczecin, Poland; renata.pilarczyk@zut.edu.pl; 2 Department of Animal Reproduction Biotechnology and Environmental Hygiene, Faculty of Biotechnology and Animal Husbandry, West Pomeranian University of Technology in Szczecin, Klemensa Janickiego 29, 71-270 Szczecin, Poland; bogumila.pilarczyk@zut.edu.pl (B.P.); agnieszka.tomza-marciniak@zut.edu.pl (A.T.-M.); beata.seremak@zut.edu.pl (B.S.); jan.udala@zut.edu.pl (J.U.); 3 Department of Animal Nutrition, Veterinary Academy, Lithuanian University of Health Sciences, Tilžes 18, LT-47181 Kaunas, Lithuania; paulius.matusevicius@lsmu.lt; 4 Institute of Animal Rearing Technologies, Veterinary Academy, Lithuanian University of Health Sciences, Tilžes 18, LT-47181 Kaunas, Lithuania; ramute.miseikiene@lsmu.lt

**Keywords:** alpaca, behavioural assessment, environmental enrichment, husbandry environment, stress responses, welfare

## Abstract

**Simple Summary:**

In recent years, interest in the welfare of alpacas (*Vicugna pacos*) has been growing, particularly in Europe, where they are becoming increasingly popular for breeding, therapy, and agrotourism. However, there is a lack of specific welfare regulations for alpacas, highlighting the need for further research and education. Alpacas thrive in environments that replicate their natural habitat, with sufficient space, appropriate shelter, and opportunities for social interaction. Proper nutrition, especially a high-fibre diet, is essential for maintaining their health. Social bonds within the herd are also crucial, as alpacas are highly social animals; disruptions in group dynamics can lead to stress and aggression. Human–animal interaction plays a key role as well: early, controlled socialisation encourages tameness, whereas excessive handling, particularly in young males, may lead to behavioural issues such as “berserk syndrome.” Grooming procedures (such as shearing and nail trimming) and training should be carried out using low-stress methods to support animal welfare. Environmental enrichment, including varied terrain, scratching posts, and foraging opportunities, helps prevent boredom and encourages natural behaviours. Monitoring behaviour is essential for identifying signs of stress or discomfort and enables timely intervention. By understanding the physical and emotional needs of alpacas, breeders can improve their well-being, resulting in healthier and more content animals. This knowledge can also inform future welfare regulations and promote more sustainable breeding practices.

**Abstract:**

A key diagnostic tool in breeding practice is systematic observation: by identifying abnormalities in natural behaviour, it can highlight early signs of reduced welfare or physical and mental health issues in livestock and other animals. The aim of this paper is to review current literature to provide a detailed analysis of the factors affecting the physical and mental health of alpacas (*Vicugna pacos*) with regard to their husbandry method. It analyses various behavioural patterns such as stress reactions to strangers, isolation and grooming, as well as social interactions (hierarchy, bonding, affiliative and aggressive behaviour), reproductive activity (courtship, copulation, partner rejection), and resting and foraging rhythms. It also discusses the influence of socialisation on human–animal relationships and the role of the environment, and its enrichment, in proper behavioural functioning. It also examines the significant role played by nutrition and the environment in ensuring alpaca welfare, and how it can be maintained during grooming and training. It pays special attention to the use of behavioural tests to assess the level of trust and tameness in alpacas. Systematic behaviour observation allows a quick response to deteriorating welfare, thus facilitating more efficient herd management and reducing the risk of more serious health and behavioural problems.

## 1. Introduction

Alpacas are currently enjoying increasing popularity as animals for breeding and recreation within the European Union. However, while EU legislation does include general regulations on animal welfare, these still lack specific conditions relating to alpaca welfare. As such, there is a growing need for research in this area, as well as greater breeder education and the creation of specific legislation.

Alpacas (*Vicugna pacos*) are domesticated camelids native to South America [1]. Thanks to their calm disposition, ease of bonding with humans, and their valuable fleece, their popularity has recently grown [2], and they are increasingly desirable as agents in animal therapy and attractions on agro-tourism farms [3]. In this regard, a familiarity with alpaca behaviour and the factors influencing their physical and mental well-being can play a significant role in ensuring their well-being.

Hughes [4] defines animal welfare as a state of physical and mental health in which animals are in complete harmony with their environment. In contrast, Mellor [5] regards it as a state that allows animals to lead lives that can be considered “of value”. Most importantly, this approach does not require the complete elimination of negative stimuli; rather, it is sufficient to minimise them and create conditions that foster positive emotional states in the animal. The author recommends that the traditional concept of the five freedoms should be changed and, instead of striving for complete freedom from suffering, pain, hunger, or thirst, a more realistic approach is to minimise these factors as much as possible. Hence, while unpleasant sensations should be reduced and their long-term impact avoided, they are to some extent inevitable; furthermore, despite their negative nature, they can serve an adaptive function by motivating animal behaviour.

Despite their positive social image, alpacas in Europe are subject to a range of environmental factors that differ from their home: weather conditions, feed quality, access to clean water, husbandry practices, and quality of care have a significant impact on their health and development, and their actual behavioural and emotional needs are often poorly understood. Prolonged exposure to unfavourable conditions can lead to a decline in immunity and overall health, and reduced breeding performance [6]. However, like with all animals, their natural behaviours, such as their resting, feeding, and social interactions, are effective indicators of potential health or emotional problems [7]. Changes in the frequency and intensity of such behaviours may indicate stress, discomfort, or physiological disorders. For example, studying the circadian rhythm of their activity can provide a valuable insight into their mental and physical condition. However, while behavioural observations can indicate initial deteriorations in alpaca welfare, they require careful interpretation: animal behaviour is strongly dependent on environmental and social contexts, and stress may derive from various sources. Therefore, it is important to combine behavioural observations with other welfare indicators, such as physiological aspects. It should also be emphasised that the knowledge and competence of the assessors play important roles in the correct interpretation.

Alpacas are herd animals that enjoy the presence of other individuals; as such, they require appropriate social relationships to maintain their emotional balance. Observing their social behaviour, including mother-offspring bonds, can allow better organisation of the breeding environment, support the development of young animals, and reduce stress resulting from isolation or conflicts within the herd [8,9].

Anxiety and stress levels can be ameliorated by appropriate early socialisation and training methods, as these will better facilitate daily handling and improve the quality of interaction with humans. Good relations with the carer will ensure a safe and comfortable breeding environment [10,11,12].

Furthermore, alpacas require regular grooming, such as shearing and nail trimming. While these procedures can be a source of stress for the animal, it is possible to reduce the associated discomfort, and thus improve their welfare, by performing them correctly and monitoring any emotional responses [13,14,15,16].

Another important consideration when keeping alpacas is environmental enrichment. An environment that is poor in stimuli has a negative impact on mental health, which encourages frustration and hastens the development of stereotypical behaviours. The keeper should aim to provide opportunities to explore, play, and interact with the environment to satisfy their behavioural needs [17].

The aim of this description is to provide a detailed analysis of the factors affecting the physical and mental health of alpacas (*Vicugna pacos*) in breeding conditions, based on a review of the available scientific literature. In particular, it discusses the importance of the relationship between humans and alpacas, with a particular emphasis on the impact of early socialisation and training methods on alpaca behaviour. It also analyses the social and sexual interactions within herds, and the bonds that form between mothers and their offspring, which play a key role in the proper development of young animals. It examines the welfare of alpacas during routine care procedures, such as shearing, nail and tooth trimming, and their impact on stress levels. The work also explores the effect of environmental enrichment on satisfying the natural behavioural needs of alpacas and discusses the importance of being able to recognise behavioural signals as a tool for assessing their well-being and emotional state.

Its findings can have a direct impact on improving the quality of life of alpacas in breeding conditions by fostering an environment that respects their biological and emotional needs. They can also serve as a base for more sustainable development and support the mental and physical health of the animals.

The corpus of articles used in the review was gathered by a search of PubMed, Web of Science, Google Scholar and Scopus using the following key words: alpaca welfare, *Vicugna pacos* behaviour, enrichment for alpacas, alpaca-human interaction, alpaca social behaviour, alpaca stress indicators, aberrant behaviour in alpacas, thermoregulation in alpacas, behavioural indicators of welfare in camelids and ethological needs of alpacas, trust, tameness, parasite infestations in alpacas, congenital and genetic defects in alpacas, limb deformities in alpacas, transport stress in alpacas, exhibition stress in alpacas, veterinary training, off-label drug use, biosecurity, transport management, quarantine conditions, health monitoring in alpacas, animal handling, stress physiology in alpacas, alpaca reproduction, and genetic selection in alpacas. The literature search was carried out by the use of the following combinations of key words and the logical operators AND and OR: “alpaca welfare” OR “*Vicugna pacos* behaviour” OR “enrichment for alpacas” OR “alpaca-human interaction” OR “alpaca social behaviour” OR “alpaca stress indicators” OR “aberrant behaviour in alpacas” OR “thermoregulation in alpacas” OR “behavioural indicators of welfare in camelids” OR “ethological needs of alpacas” OR “trust” OR “tameness” OR “parasite infestations in alpacas” OR “congenital and genetic defects in alpacas” OR “limb deformities in alpacas” OR “transport stress in alpacas” OR “exhibition stress in alpacas” OR “veterinary training” OR “off-label drug use” OR “biosecurity” OR “transport management” OR “quarantine conditions” OR “health monitoring in alpacas” OR “animal handling” OR “stress physiology in alpacas” OR “alpaca reproduction” OR “genetic selection in alpacas”. In some cases, to narrow the results, the queries were combined with the keyword *alpaca* using the ‘AND’ operator. The query syntax was adapted to the available advanced search options depending on the database.

The information gathered was based on material from original and review articles, as well as books and reports.

The initial search of PubMed, Web of Science, and Scopus yielded 1134 original results. After removing duplicates between the databases, 748 publications remained. A review of the titles and abstracts narrowed this total to 312 publications, of which 112 were rejected as being unrelated to the topic, leaving 200 articles.

The Google Scholar search yielded a larger number of publications, but a significant proportion of these were of limited scientific value, including preprints and theses. After discarding these, 172 items remained. Of these, 38 were excluded due to lack of access to the full text, and 21 for methodological reasons, resulting in 113 eligible publications.

In total, 313 publications were shortlisted for further analysis. Of these, 122 key items were selected for final review.

The selected publications were divided into the following areas: (1) alpaca (*Vicugna pacos*) welfare in the breeding environment, (2) the influence of nutrition on alpaca welfare, (3) human–alpaca relationships, (4) social and sexual interactions, (5) the bond between mother and offspring, (6) alpaca welfare during grooming, (7) alpaca welfare during training, (8) enrichment of the alpaca keeping environment.

The final articles for inclusion were selected on the basis of the following inclusion criteria:

— Articles must be related to the welfare of alpacas or aspects related to their husbandry, i.e., behaviour, feeding, social interaction, relationship with humans, grooming, training, or enrichment of their environment.
— Original scientific studies, literature reviews, and chapters that present reliable data on the topic under study.
— Works that provide full access to allow a thorough analysis of the methodology and results of the study.

The collected information was analysed and organised thematically. The findings are presented in relevant sections, allowing for comprehensive coverage of the topic.

## 2. Influence of the Housing Environment on the Welfare of Alpacas (*Vicugna pacos*)

### 2.1. Living Conditions

An important aspect of alpaca welfare is providing adequate housing conditions, which is particularly important in the variable climate of Europe. Alpacas are typically kept in year-round facilities, such as livestock buildings or sheds, whose primary function is to provide protection from adverse weather conditions. They should also have constant access to shelter in the paddocks in case of rain, wind, or excessive sunlight. It is also very important that adequate space be provided for alpacas. Furthermore, regardless of the size of the herd, alpacas should be able to observe each other [18] (Table 1).

In Europe, alpacas are mostly kept in semi-intensive systems. The animals are provided with shelter in purpose-built facilities where they are guaranteed suitable environmental parameters, while also allowing them to regularly use separate grazing areas.

Simple, uninsulated wooden structures are particularly suitable for alpacas as they provide good ventilation and access to daylight, encourage natural behaviour, and reduce the risk of respiratory diseases. The alpacas should also be provided with proper bedding and shelter from adverse weather conditions. Using a layer of sand covered with straw as a substrate creates a dry, soft, and well-insulating environment, which benefits thermoregulation and reduces the risk of limb injuries; the use of concrete surfaces without proper bedding can lead to hypothermia and increase the risk of health problems, especially in winter [19] (Table 1).

The living space should be designed in accordance with the number of animals, age, sex, and physiological needs. Housing should include separate pens to reduce any stress which may be caused by poor social interaction. Pregnant females, and those with young, are best kept in a separate, quiet area, which provides greater comfort and reduces the risk of behavioural disorders or injuries. Juveniles over the age of six months should be moved to separate pens; this is especially important for young males, who may begin to exhibit territorial and competitive behaviour at the age of one to two years. Breeding males are best housed in pairs in separate pens or even a separate building to avoid conflicts that could compromise their health [18].

It is recommended that at least 2 m^2^ should be provided for each alpaca to allow them to move freely, lie down, and adopt natural body positions. Each pen should include hay feeders, concentrated feed troughs, and water containers, and the animals should have access to clean water and high-quality feed [18] (Table 1).

### 2.2. Pasture

During the summer, when alpacas are mainly at pasture, special attention should be paid to the risk of overheating. Coming originally from cooler, high mountainous areas, they are sensitive to high temperatures, and so shade (e.g., through trees or sheds) and constant access to fresh water should be provided, as these conditions have a direct effect on reducing heat stress and increasing welfare [18]. Alpacas possess a number of special zones in the body adapted for the dissipation of heat, known as windows; these are located inter alia on the abdomen, in the groin area, and on the inside of the thighs, where the skin and the hair are noticeably thinner. These areas, covering about 20% of the body surface, play an important role in thermoregulation by allowing the body to cool effectively [20] (Table 1).

Alpacas use various behavioural strategies to regulate their body temperature, such as changing their posture to control the size of the thermal window. They also seek out shaded or airy areas and use water to cool down [20,40] (Table 1).

Alpacas often use areas designated as latrines to address their physiological needs [21,22]. Sharing a defecation site indicates a high degree of social organisation and low levels of group anxiety, and is particularly evident among females, who queue up to relieve themselves. Such orderly behaviour is often a sign of well-being and stable relationships within the herd (Table 1).

## 3. Impact of Nutrition on Alpaca Welfare

A cornerstone of ensuring good health and welfare among alpacas is meeting their specific nutritional needs and providing a well-composed diet.

As alpacas originate from regions where access to food is limited, their digestive system and metabolism are well-adapted to a diet low in nutrients but high in fibre. Also, while the alpaca is able to temporarily draw on reserves stored in various tissues in times of scarcity, prolonged use of these reserves without replenishment can lead to serious health problems and even death [19] (Table 1).

However, in Europe, alpacas are most often kept in grazing systems whose forage quality is subject to seasonal variation, resulting in varied growth rates and health status [41,42]. Their nutrition should be based on roughage such as grass and hay, which should make up about 80–90% of the total diet. This can be supplemented with root vegetables such as carrots and beetroot, or feeds such as beet pulp or alfalfa [18]. The digestive system of alpacas is not adapted to digest large amounts of simple sugars and starches, so an excess of concentrated feeds in their diet may result in digestive disorders; such feed should be used in moderation to avoid sudden changes in dosage. A good solution is pelleted mixes, which act as carriers of micronutrients and vitamins; however, it is important that they contain not only cereals, but also other fibre-rich ingredients such as bran, alfalfa, soya hulls, or beet pulp. Compared to other livestock, alpaca feeds should have a lower content of starch and simple sugars [18] (Table 1).

Pasture organisation also plays an important role in ensuring welfare. Fencing should be solid and sufficiently high, at least 1.4 m for females and 1.5 m for males; in the case of the males, an additional board or beam should be added on top, especially if they are in close proximity to females. Such protection prevents escapes, injuries, and uncontrolled contact, which can cause stress and aggression [18].

The maximum recommended stocking density is 15 alpacas per hectare of pasture, if the main source of food is grass [18,19]. This would allow sufficient food and space for the animals to move freely, which encourages their natural behaviour and reduces competition for food (Table 1).

Alpacas, being camelids, have soft soles that do not damage turf. Their foraging method involves gently nibbling on plants without tearing them from the roots, which helps to keep the pasture in good condition [19].

To ensure balanced grazing and prevent conflict and excessive competition for resources, the pasture should be divided into separate areas for different groups of animals (e.g., males, females, juveniles). This would also reduce stress due to hierarchy and lower the risk of injury, especially to young animals. At least half of the area should remain in reserve to allow vegetation to regenerate. The alpacas should be regularly moved to a new area when the grass height falls below 5 cm as part of rotational grazing. If the pasture is overgrazed, hay feeding should be introduced, and this should be provided in feeders rather than directly on the ground [18].

Nutrient deficiencies adversely affect the immune system and welfare of the animals and reduce their fleece quality and fertility. A study by Rios et al. [43] on alpacas living at high altitude found they spend most of their time (78%) grazing, with the remaining time being divided between walking (7%), ruminating and resting (4%), or drinking, sand bathing, and defecating (11%). Analysing these behaviours provides a better understanding of the needs of alpacas and the factors affecting their welfare.

The vitamin D levels of alpacas vary with the seasons, peaking at the end of summer [44] and reaching the lowest level around the end of winter and beginning of spring [45]. However, due to limited sunlight and insufficient dermal synthesis, vitamin D deficiency is a common problem for those in temperate climates, such as Poland; as such, adults require vitamin D supplementation during winter, and juveniles need it throughout the year to prevent rickets [19]. Furthermore, selenium supplementation may be necessary in Se-deficient areas [23] (Table 1).

Like most herbivores, alpacas devote a large part of the day to foraging and chewing energy-poor but fibre-rich forage. Foraging also has a social dimension: it is typically initiated by one individual and triggers similar activity in other herd members [46]. However, unlike many other ruminants, alpacas mainly nibble on low-growing grasses using their bicuspid upper lip and lower incisors: they do not use their tongue to manipulate forage, which only occasionally protrudes outside the mouth [24,47]. As such, they rarely use salt licks, and they require alternative sources of vitamins and minerals, such as supplements, which can be mixed with concentrated feed [24]. Failure to provide suitable supplementation can adversely affect health and welfare by increasing the risk of deficiencies (Table 1).

Adequate watering is very important for the condition and welfare of alpacas. The animals should have unlimited access to fresh and clean water, which should be replenished regularly. Their water requirements can increase by as much as six litres per day per animal in summer, especially for lactating females, and the water should be changed more frequently in hot weather to keep it cool and encourage drinking. The water should also be provided in open tanks, as alpacas prefer to drink with a slightly open mouth. Drinkers with a float that maintains a constant water level are accepted, while those that require pressing a lever are not [12,18] (Table 1).

Another important consideration is the hierarchical structure of the herd. Alpacas in a lower position may face difficulties in accessing food and water, especially when the dominant animals control their access. This situation can lead to stress, reduced physical condition, and health problems. Therefore, to ensure equal access and reduce rivalry within the flock, it is important to provide an adequate number of feeding and watering areas.

## 4. Human–Alpaca Relations

An important part of alpaca husbandry is their relationship with humans, whose nature is largely shaped by the quality and frequency of contact between the keeper and the animal, especially during the first stages of life. Alpacas show a strong ability to recognise people, which allows them to be handled easily in everyday settings. However, in situations involving contact with strangers, such as during physical examinations, they can show signs of stress, such as freezing, which reflects their high behavioural sensitivity [11].

Windschnurer et al. [11] indicate that the behavioural responses of alpacas are significantly influenced by the attitudes of their caregivers. Animals whose caregivers were positive about tactile contact and verbal communication showed fewer stress reactions and were less likely to attempt to escape from humans. Hence, the quality of the human–animal relationship is fundamental to the welfare of alpacas and their use.

When properly tamed, alpacas do not usually show aggression towards humans, and spitting is rare [10]. Caution is advised regarding intense physical contact with very young individuals. In these animals, excessive stroking or cuddling can lead to mis-imprinting and the perception of humans as individuals of the same species; this can result in inappropriate behaviour, such as kicking, biting, and even charging, directed at humans, similar to how alpacas compete with each other in a herd. Such behaviour is often labelled as berserk male syndrome or aberrant behaviour syndrome; however, this problem does not only affect males: mis-imprinting phenomena can lead to females rejecting male alpacas during the heat period. If the female considers humans as her partners, she may not accept the courtship of males of her species, making reproduction difficult or impossible [31,35,36] (Table 1).

### 4.1. Socialisation

Over-socialisation of young male alpacas with humans can also lead to behavioural disorders later in life [25], a phenomenon sometimes referred to as berserk syndrome, most commonly observed in alpacas between one and three years of age. Its occurrence has been attributed to imprinting resulting from inappropriate levels of human contact, i.e., too much time with humans compared to their own herd, in the first weeks of life, and is particularly common among bottle-fed individuals [37]. The animals become excessively attached to humans, which at a later age, especially after sexual maturity, can result in aggressive or sexual behaviour towards humans [48]. In males, the consequences are sometimes so severe that euthanasia is necessary, while the females usually show milder symptoms, such as increased nervousness or a tendency to defend themselves by spitting [37]. It is therefore advisable to be cautious when dealing with young alpacas, especially when feeding them artificially, to minimise the risk of such undesirable behaviours (Table 1).

Young male alpacas can be a challenge for novice keepers. If the owner misinterprets and inappropriately rewards animal behaviour (e.g., through excessive petting), the alpaca may be encouraged to invade their personal space. The animal may attempt to dominate the human, mimicking natural struggles for position in the herd. While this behaviour may initially only be disruptive, it can become more serious over time [36].

However, a study of early socialisation by Miranda-de la Lama et al. [12] found that animals that were regularly stroked and had frequent contact with humans in early life showed greater submissiveness and less resistance when guided. In contrast, limited tactile contact in later life stages significantly correlated with handling difficulties and more frequent avoidance of human contact (Table 1).

Therefore, it is important to maintain an appropriate balance when associating with young alpacas and to avoid excessive anthropomorphisation, which can lead to disturbances in their behavioural development. Rather, young alpacas should be allowed controlled and restrained contact with humans, while also associating with other alpacas. This approach favours the formation of correct social behaviour and reduces the risk of undesirable behavioural reactions in adult life.

### 4.2. Behavioural Testing

The relationship between humans and alpacas can be assessed using the same behavioural tests performed for animal species, which evaluate how an animal reacts to the presence or action of a human. One of the basic approaches is an interaction test, in which the animal is free to approach a stationary human [7] or a human approaches a stationary animal [39]. Both methods determine the level of trust or distance in the human–animal relationship [49]. These tests are usually carried out in a confined space, such as a pen or enclosure, and the observation itself takes only a few minutes [39]. Taylor and Davis [50] found that the responses of alpacas vary depending on whether they are interacting with a familiar person, suggesting that animals can discriminate between humans and build different types of relationships with them. An important indicator used for assessing the degree of tameness of an animal and the quality of contact with humans is the escape distance, i.e., the minimum distance at which an animal starts to move away from a human [38] (Table 1). Its value is mainly influenced by previous interactions between the alpaca and humans [51]. Animals with an escape distance of zero, i.e., those that allow themselves to be touched and show no signs of fear, are considered fully tame [52]. In the literature on alpaca and llama behaviour, the quality of the animal–human relationship, and the way the animals respond to contact with their keeper, are typically described using the terms trust and tameness. In alpacas, trust can be defined as the degree to which the animal allows close contact and manipulation by humans, without exhibiting strong avoidance or stress behaviour; this can be manifested by inter alia the animal tolerating touch, making contact on its own initiative, and behaving calmly during routine grooming procedures. Tameness, on the other hand, is understood as a general level of submissiveness and reduced fear towards people; it is manifested as shorter latencies when approaching new objects or humans, fewer avoidance behaviours (e.g., baulking, escape attempts), and ease of handling [11,53,54,55].

However, as long as they have been properly prepared from a young age, i.e., they can accept the presence of humans but not treat them as their equal companion, alpacas are easily capable of taking part in activities with humans. Indeed, those that can function in a human environment without undue stress and without attempting to dominate do well in a variety of work settings. However, to ensure the safety and well-being of both animals and humans, the carer should have a good understanding of their natural needs and how to communicate [29].

The degree of tameness of an alpaca, its relationship with humans, and its general welfare can be determined by the regular use of behavioural tests. When an animal responds calmly to the presence of humans and does not exhibit avoidance behaviour, it is most likely enjoying an environment that meets its physical and emotional needs, which is an indispensable element of their welfare; among alpacas, this is clearly indicated by a high level of trust towards the handler, a shorter escape distance, and no signs of stress.

## 5. Social and Sexual Interactions

Kapustka and Budzyńska [3] indicate that the social repertoire of alpacas includes both submissive signals and aggressive behaviour. Submissiveness is expressed by lowering the front part of the body, flattening the ears, and raising the tail. In contrast, warnings and threats are indicated by the alpaca stretching the neck out and laying the ears flat, with the head forming a continuation of the neck or an angle of approximately 120°; these signs are typically expressed when two individuals stand next to or opposite each other. Sounds resembling snorting or sneezing are then emitted, often followed by spitting: a defensive act in which the animal ejects saliva, often mixed with stomach contents. Aggression is manifested by a raised head, straight neck, open mouth, and the tail along the line of the back. Other signs of tension include chasing with the neck lowered and tail raised, and group play involving romping and mock fighting.

### 5.1. Hierarchy

Being a herd species, alpacas exhibit a distinct social hierarchy based on dominance. In the herd, a male plays the role of the dominant individual, while females and young submit to his position. There is also a hierarchical differentiation within the female group, often related to age, with older individuals occupying higher positions than younger ones. The lower individuals show signs of submissiveness towards the dominant ones [46]. The authors note that alpacas exhibit distinct social behaviours, including both conflict and affiliation responses, and warning responses were observed, often accompanied by characteristic spitting, in contentious situations, such as competition at the trough or invasion of personal space. In such cases, the lower-ranking individual usually gives way, thus avoiding direct confrontation. Nevertheless, alpacas are gregarious animals, and as such can exercise affiliative behaviour by forming strong social bonds; individuals in close relationships spend most of their time together, feeding and resting in each other’s company. As noted previously, remaining in a familiar group appears to have a positive effect on well-being and welfare. Interestingly, alpacas engage in quiet purring as an example of affiliative behaviour, which serves to maintain contact between individual members of the group.

### 5.2. Social Bonds and Behaviour

Pollard and Littlejohn [26] confirm the presence of strong social bonds among alpacas. They report that isolating alpacas from the rest of the group in a confined space resulted in symptoms of severe stress: the animals exhibited increased mobility, anxiety in the form of intense head movements, reduced appetite, and accelerated heart rate.

When faced with danger, alpacas display strong social behaviour. The individual that first detects danger alerts the herd with a piercing sound, while assuming a characteristic posture (i.e., with raised tail and ears) facing the threat. The rest of the herd instinctively gather together, seeking safety in numbers [46] (Table 1).

### 5.3. Sexual Behaviour

The sexual behaviour of alpacas requires complex interactions between males and females and involves both physiological and behavioural factors. In males, reproductive behaviour includes the Flehmen response, a characteristic backward bending of the head, slightly open mouth, ears pricked, tail lowered, and intense sniffing [46]. The act of copulation is preceded by a courtship phase, during which the male usually chases the female, trying to force her to lie down. Although some females showing readiness to reproduce assume a lying position immediately, most initially avoid intercourse, forcing the male to stand on his hind legs and exert pressure on her hindquarters [15]. After accepting the male, the female lies down, and the male assumes a characteristic semi-sitting position and begins intromission. Unlike many other livestock species, the pelvic movements of alpacas are relatively gentle. During copulation, the male makes specific sounds, called orgling, which are most likely caused by vibrations of the soft palate [15].

The behaviour of females is strongly dependent on their reproductive readiness. Females unwilling to mate often react by running away, kicking, spitting, or adopting a threatening posture. After ovulation, females that are no longer receptive may explicitly reject the male by spitting at him, a behaviour known as spitting off [56].

Pollard et al. [57] report that the sexual activity of alpacas is significantly influenced by both the season and the prior reproductive experience of the female. Seasonally, females exhibited significantly more avoidance and defensive behaviours, such as kicking, spitting, and threatening, in spring than in autumn. In addition, in spring, the period preceding copulation was longer, while copulation itself was shorter, lasting a mean period of 14.1 min compared to 18.5 min in autumn. Reduced readiness to reproduce in spring also translated into a lower chance of successful fertilisation. Regarding prior experience, inexperienced females entering their first reproductive season were more likely to exhibit escape and kicking behaviours. They were less likely to react by spitting or threatening, which are typical of more assertive forms of defence. Despite these noticeable differences in behaviour, mating success (i.e., the percentage of successful inseminations) did not differ significantly between females that had already given birth and those without previous pregnancies.

### 5.4. Aggression

Aggression between male alpacas can be very intense, especially when it concerns competition for females; this can lead to physical confrontations such as chest bumping, neck wrestling, and attempts to knock the opponent over, as well as bites to the neck, limbs, scrotum, and head. They are also more likely to spit stomach contents and make loud, roar-like sounds. In general, spitting is an expression of strong emotional arousal; it is often preceded by warning signals, such as air being expelled from the mouth or small amounts of saliva, sometimes mixed with food, being spat out. These clashes can lead to serious injuries, especially if the males have their fangs intact. The dominant male actively defends his herd by chasing away intruders [15,46].

Uncastrated male alpacas display strong territorial instincts. In practice, this means that they are reluctant to share space with other males, and contact can lead to aggressive behaviour and regular conflicts. Such fights are often violent and can result in serious injuries, especially around the neck and genitals. As such, when males are housed together in confined spaces, such as in a stable, the subordinate can experience prolonged stress due to the lack of any escape route, unlike in the wild. Groups of males that have been castrated at a young age are much less problematic; they are calmer and less likely to clash with each other. Therefore, a good solution in breeding is to keep adult, uncastrated males separate, while still providing visual contact with other individuals. This can help them maintain a sense of control over their environment without the need for physical confrontation [29] (Table 1).

Kapustka and Budzyńska [46] note that female alpacas rarely engage in physical conflicts, and their aggression is usually limited to spitting at each other and snorting. Violations of personal space, for example, by humans approaching less domesticated individuals, may result in escape behaviour.

## 6. Relationships Between Mother and Offspring

The relationship between a female alpaca and her newborn (cria) has a strong influence on its survival and development in the first hours of life. Although this bond may seem subtle compared to the behaviour typical of other mammals, it is clear and based on characteristic mechanisms of recognition and communication.

After birth, which usually takes place in the morning (7:00–13:00), the young is left to dry itself, stand up, and find its mother [12]. The first attempts to stand are made about half an hour after birth. Although these initial movements are initially uncertain and poorly coordinated, it is important that the cria performs them unassisted, as this will help strengthen its muscles and teach it to keep its balance [58].

The mother does not exhibit classic nurturing behaviours such as licking newborns or eating the placenta [59], but remains close by, watching closely and protecting them from strangers.

One of the most important elements of bonding is scent recognition. Mothers can identify their offspring very quickly by their smell, and unknown young are firmly rejected, most often by spitting or adopting a defensive posture [27]. Vocal communication also plays a very important role in bonding, with the mother and cria exchanging quiet, characteristic sounds, which help them to recognise each other and build a relationship [8].

Although the female does not usually initiate contact with the newborn, she gains interest when it starts suckling. It is very important that the cria starts feeding within the first hour of life, as colostrum is only produced for about 24 h after birth. Subsequent suckling occurs regularly, on average every 30 min during the first four hours [60,61]. In the following days, the young stays close to its mother, who closely monitors its activity. Although physical contact is limited, the mother maintains a constant presence and strengthens their relationship and sense of security through joint movements and vocal interactions.

This bond is put to the test at weaning. When the young are separated from the mother, they experience stress, as indicated by an increase in cortisol levels; however, after rising during the first three days after weaning, these levels soon return to baseline values by day five [9]. The temporary increase in stress indicates the importance of emotional bonding in the well-being of the young animal. Separating the crias from the rest of the herd, for example, by taking them home, can lead to the development of behavioural disorders [62].

Young male alpacas exhibit playful behaviours such as jumping, chasing, simulated fighting, and interaction with objects; these build the coordination, strength, and social competence necessary for later reproductive interactions. The males do not reach full reproductive capacity until two or three years of age, when the foreskin becomes detached from the glans penis. Before this time, while the foreskin remains partially attached, these behaviours allow the exercise of motor and social skills without actual sexual activity. The early play supports correct development and improves physical and behavioural skills [63].

## 7. Health Problems Experienced by Alpacas and Their Impact on Welfare

Before discussing the health issues faced by alpacas, it should be stressed that the tendency for non-veterinarians to diagnose and treat alpacas poses a significant threat to their welfare, particularly in North and South America.

### 7.1. Parasite Infections

A lack of specialised training in camelid health and welfare often results in inappropriate use of drugs, which can promote resistance to antiparasitic agents. For example, a study carried out in Australia identified widespread resistance to the antiparasitic agents mainly used by alpaca owners [64]. As such, there is a need for greater specialised knowledge among keepers and those administering drugs to animals.

When treating alpacas, one of the most serious problems concerns the use of chemotherapeutics in off-label regimens and at unsuitable doses. It has been frequently observed that dosage recommendations for sheep or cattle are copied for alpacas; this can result in suboptimal doses, which favour the selection of resistant strains [64] and encourage the persistence of high levels of gastrointestinal parasite infestation in alpaca populations [65,66].

Chronic parasitic infections are clinically manifested by anaemia, weight loss, diarrhoea, and a general deterioration in the condition of the host [67,68]. These symptoms result in pain, stress, and discomfort, which is in clear contradiction to the fundamental principles of animal welfare. Hence, it is necessary to provide adequate veterinary training and implement integrated parasite control strategies to protect the health and welfare of alpacas.

### 7.2. Congenital and Genetic Defects in Alpacas

As successful breeding is highly dependent on reproductive performance, it is essential to monitor reproductive disorders and analyse their causes [69]. Among alpacas, around 33 to 40% of covered females do not produce offspring, 7 to 25% of pregnancies result in abortion, and 9 to 12% of young die before six months of age [70,71,72,73]. These reproductive problems can be attributed to a limited gene pool, lack of mating control, and inbreeding, which encourage the perpetuation of recessive mutations and the appearance of genetic defects [59,74].

Most alpaca breeding programmes focus on fleece quality by improving fibre quality and quantity, reducing fibre diameter (FD) and increasing fleece weight (FW), despite the low positive genetic correlation between these traits (0.1–0.35). Other objectives include increasing fleece density, fibre homogeneity, reducing the ‘scratch factor’, and selecting for morphology and appearance [69,75,76,77,78]. Selection takes place in several stages: individuals with phenotypic defects may be eliminated at birth, others through evaluation at weaning, fibre quality control, and sire evaluation can be performed using the BLUP model, or a combination of visual and laboratory evaluations of fibre [79].

Congenital and genetic defects have a significant impact on alpaca welfare. Many demonstrate abnormalities of the head, craniofacial structures, dentition, or heart, which prevent food intake and impair respiration and cardiovascular functions, leading to stress, pain, and reduced survival [80,81,82,83,84,85]. In addition, reproductive defects and chromosomal aberrations, including ovarian and testicular hypoplasia, cryptorchidism, hypospadias, or XX/XY chimerism, result in greater infertility and limit natural social and reproductive behaviour [80,85,86,87,88,89,90]. As such, there is a need for more accurate genetic control and reproduction selection to improve alpaca welfare and breeding performance (Table 2).

### 7.3. Limb Defects

Limb deformities have a direct impact on welfare. Above-ankle and knee valgus (angular limb deformities) limits mobility, causes joint pain, and increases the risk of injury [91,92]. Syndactyly (fused toes) impedes mobility and limits limb function [93], as does polydactyly (extra toes), which also interferes with balance [93]. Arthrogryposis (stiffness of the limb joints) leads to restricted mobility and difficulties in walking and food intake [80]. Furthermore, alpacas are at risk of bone mineralisation defects, such as rickets and osteomalacia, which can result in limb deformities, pain, and an elevated risk of fracture [93]. Trauma or poor footing can also cause limb deformities resulting in limited mobility and chronic degenerative changes in the joints [94] (Table 3).

## 8. The Five Domain Model

Alpaca welfare can be interpreted according to the Five Domain Model, i.e., Nutrition (assessment of Body Condition Score—BCS, micronutrient levels and foraging time), Environment (body temperature, microclimate parameters, access to shade and water), Health (cortisol concentrations, heart rate variability, dental and nail condition), Behaviour (aggression levels, social interaction, proper suckling of young) and Emotional/Psychological state (reactions to the caregiver, cooperation, stress levels), with each domain defined by behavioural and physiological indicators. The interpretation of the model can be used to implement appropriate management strategies, including diet, environmental conditions, preventive health care, and gentle handling methods (Table 4).

## 9. The Welfare of Alpacas During Grooming Procedures

### 9.1. Shearing

In everyday practice, it is essential that alpacas receive routine grooming care, which plays an important role in maintaining their health and well-being. One basic procedure is shearing, which is usually carried out once a year; this not only provides valuable fleece, but also prevents overheating and heat stress during the summer months. Indeed, Navarre et al. [30] report that alpacas that have not been sheared are more sensitive to heat stress than those that have [30].

No matter how used they may be to humans, alpacas still find shearing stressful, especially when the conditions are not adequate. Alpacas in stressful situations may exhibit various forms of defensive reactions. These include behaviours such as spitting, biting, kicking, and vocalising (e.g., squeaking, howling, snorting, or grinding their teeth), and performing more passive reactions such as suddenly lying down on the ground or freezing in place [11,31] (Table 1).

A very important factor affecting animal welfare during shearing is the method used to restrain them, which can significantly affect their stress levels [13]. An analysis of clinical indicators and cortisol in saliva showed that alpacas tolerated immobilisation better when standing than lying down, both on a mattress and on a shearing table; however, no such differences were found in the levels of cortisol metabolites in faeces. These differences are probably due to the forced lying position of the animals and the fact that their limbs are immobilised with ropes, which gently but firmly stretch them. The authors therefore propose that the alpaca should be immobilised in a standing position, assuming that it tolerates contact with humans well and does not display stress. This form of shearing is the least stressful for the animal. However, for alpacas that react with severe stress, shearing them in a lying position, i.e., on a mattress on the ground or on a tilting table, is a safer solution that minimises the risk of injury to both the animal and the operators [13] (Table 1).

Immediately after shearing, alpacas tend to reduce their food intake and rumination, remain standing more often, and increase their social interactions. In stressful situations, they may vocalise, moan, scream, and even growl. Interestingly, a study by Waiblinger et al. [14] found that animals sheared in a standing position screamed less frequently and that the screaming usually stopped after the shearing machine was turned off; this may indicate that stress is associated with the sound of the machine and immobilisation (Table 1).

### 9.2. Hoof Correction

Another important procedure is nail trimming, as overgrown nails can lead to mobility problems and even inflammation. Alpacas are raised on pastures, which differ significantly from their natural environment. Depending on the conditions, their nails require regular care at least three or four times a year. However, the rate of nail growth may vary, with the nails of alpacas with lighter coats growing faster and often thicker than those of darker animals. Neglected nails can become deformed, leading to weight loss due to lameness, reduced mobility, and difficulty moving while grazing. Alpacas accustomed to regular grooming and nail trimming are relatively easy to handle. Generally speaking, holding the animal gently but firmly is more effective than restraining it by force (e.g., in a special pen) and causes less stress. It is recommended that one person gently restrains the alpaca while the other carefully trims its nails. It is best to use scissors with straight blades, special clippers, or shears such as those used for trimming hooves. The nails should be trimmed flush with the soft pad, taking care not to damage the living tissue and cause bleeding [15,16,105] (Table 1).

However, this procedure can be problematic, especially for animals that are not accustomed to being touched or having their limbs manipulated. For a number of reasons, alpacas naturally show anxiety or resistance during nail trimming: their legs are a means of escape and defence, males often bite each other’s legs during fights, and females may perform similar gestures for reproductive purposes. Also, previous bad experiences with improper handling of the limbs can increase stress and panic during the procedure. Therefore, it is important to gradually accustom the animal to being touched and having its nails trimmed. Again, animals familiar with this procedure are usually easier to handle [15,16,32,101].

### 9.3. Tooth Correction

In some cases, dental correction may also be required, especially when there is excessive growth or malocclusion, i.e., where the teeth are too long and protrude beyond the outline of the muzzle, which can make eating difficult [33,34]. Also, crooked or misaligned teeth can prevent the animal from chewing: such deformities can cause damage to the oral mucosa, leading to abrasions or ulcers (Table 1).

Dental correction procedures can be stressful and unpleasant, so it is important that they are approached with great care to ensure the well-being of the alpaca. Firstly, the animal should be properly prepared for the procedure, such as familiarising them with touch and manipulation in the mouth area beforehand. It is also important to use gentle immobilisation techniques that minimise the feeling of threat and allow the alpacas to maintain their balance, as the stress associated with forced immobilisation can cause negative reactions such as aggression or escape attempts, complicating the procedure and affecting well-being [102].

## 10. Ensuring Well-Being During Training

Currently, alpacas are valued for their gentle disposition and ability to learn, which makes them increasingly popular in animal-assisted therapies (alpaca therapy) and agrotourism. It is hence important that they learn to accept a halter and move calmly on a line.

Nowadays, increasing importance is being attached to the proper training of alpacas to facilitate their daily care. Traditional methods based on coercion or intimidation often lead to increased aggression, which can pose a particular threat to children and inexperienced handlers [101]. In extreme cases, when behaviour becomes uncontrollable, euthanasia may be necessary. In contrast, positive reinforcement techniques have proven effective in teaching basic activities such as leading, shearing, loading, and transport, and responding to veterinary procedures [31]. Research indicates that gentle training methods, such as soft touches, a calm tone of voice, and gradual familiarisation, significantly reduce stress levels in camelids and make their behaviour more predictable [106]. Although alpacas that feel fear initially avoid contact with humans, extinguishing their stress response builds lasting trust [107].

A recommended method for working with alpacas is Camelidynamics, which is based on the principles of positive interaction and observation of animal body language. The concept involves creating harmonious relationships with the animal, allowing the alpaca to feel comfortable, accept touch, and be led without resistance [31].

Training can begin as early as six months of age, after the animal is familiarised with human presence and touch [11]. Below six months of age, excessive contact between humans and alpacas can lead to the development of Berserk syndrome, particularly when the animal is being bottle-fed and has limited contact with the herd [25,37].

Training begins in a small pen (catch pen) with at least two alpacas to reduce stress, with the handler using a long training line and a whip-like stick [31]. The line is placed around the neck using the stick, and this is then gently transformed into a collar. The trainer teaches the animal to grip under the jaw and behind the ears, using a bracelet to safely immobilise it, and then puts on a halter. The halter must be fitted to the alpaca, with an adjustable noseband and headpiece [31].

It is best to use a narrow, fenced-in space when teaching the animal to walk on a lead. The animal should not be pulled; in fact, the line should be loosened as a reward for each step. Once these basic skills have been mastered, the alpaca can be accustomed to different surfaces, rooms, stairs, lifts, and transport. Food helps to overcome fear and build positive associations.

The next stage is to desensitise the limbs, as alpacas have a natural kicking reflex [3]. The trainer should begin at the neck or back and gradually move towards the legs, using food where necessary; however, even after desensitisation, it should not be assumed that the animal will not kick. Nevertheless, desensitised alpacas can be taught to offer their legs for grooming. Camelidynamics can be supplemented with positive reinforcement training, e.g., with a clicker [3]. Alpacas can also learn simple tricks, such as ‘kisses’ or coming when called.

The effectiveness of training is very much influenced by the approach taken by the handler; when performed correctly, the alpaca is more likely to adopt a friendly attitude in stressful situations, characterised by less vocalisation [11]. A particularly important role was found to be played by early socialisation: frequent, gentle physical contact in early life improved docility and cooperation, while its absence at a later age increased handling difficulties. To improve human–animal relations, it is important to gradually accustom animals to the presence of humans, train staff in proper handling, and design pens that allow for safe group movement [12]. Anderson [108] recommends the use of a pen system, in which the entire herd is first gathered and the necessary individuals are later isolated; indeed, Fowler [105] notes that attempting to capture a single animal without first gathering the group often results in stress and escape.

It is important to be able to recognise the behavioural signals given by alpacas, as this can indicate whether they feel safe or are experiencing anxiety. Signs of stress may include frequent spitting, nervous movement, turning the head away, bristling fur, raising the tail, excessive alertness, or a stiff body posture [12]; they may also signal severe anxiety with loud cries and alarming high-pitched sounds, referred to as whistles or neighing [109]. In contrast, a relaxed posture, calm chewing, lying in a bridge position, and natural, fluid movements indicate calmness and a sense of security. An alpaca may express interest in its surroundings by raising its ears, focusing its gaze on a stimulus, murmuring quietly, or cautiously approaching a new object [12]. Knowledge of these signals is extremely helpful in assessing the emotional state of the alpaca and in appropriately adjusting environmental conditions and interactions.

Although stress levels can be assessed by measuring hormone levels, this method can be counterproductive in the case of alpacas, as the blood collection procedure itself is stressful enough to distort results. Therefore, increasing attention is being paid to alternative techniques that do not cause discomfort, such as those involving the collection of other materials such as saliva, milk, or fur [103]. Saliva, in particular, is considered a valuable source of information, as it contains the biologically active form of stress hormones [104], which can be very useful in assessing the current emotional state.

Generally speaking, the use of short, regular training sessions significantly reduces anxiety and increases acceptance of new experiences by the alpaca [110]. A routine that includes leading, tying, and touching sensitive areas (neck, legs) improves cooperation with animals during the productive period [12].

## 11. Alpaca Welfare During Transport and Exhibitions

### 11.1. Transport

Animal transport is a significant stressor that can affect both welfare and physiological health. Juveniles are particularly vulnerable, and often experience anxiety, reduced appetite, and lowered immunity when being transported [111]. Transport can increase the risk of stress, injury, and disease when performed under inappropriate conditions, resulting in poorer health and well-being. Therefore, even short journeys require careful planning and trained handling.

Safe transport requires a suitably adapted vehicle with sufficient space, a non-slip floor, and good ventilation. Alpacas should also be transported in groups, and feeding should be limited to hay during the journey, with the rest of the food being provided during stops. Noise, sudden vehicle movements, and extreme temperatures should be avoided. The condition of the animals should be monitored, and rest and watering breaks provided.

The welfare of the transported alpacas is subject to stress at every stage, from preparation, to loading and transport itself, to unloading and contact with the new environment; this stress is exacerbated when the animals have to wait in adverse conditions [112]. Even short transports of up to four hours have been found to alter physiological indicators of stress, such as muscle glycogen levels and muscle metabolism [113]. Interestingly, a seven-day rest period did not appear to increase glycogen reserves or influence final muscle pH after a four-hour transport; this suggests that short transports also have some long-term effect on muscle metabolism. In another study, a 30 min transport caused a transient increase in cortisol levels, but no significant changes in heart rate or observed behavioural traits were noted. After a four-hour rest, cortisol levels returned to baseline values, indicating a rapid normalisation of the hormonal response upon the cessation of transport stress [114].

Hence, even short journeys can cause transient stress, and this is mostly regulated after a rest period, both at the hormonal and metabolic levels. Therefore, minimising the negative impact of transport requires appropriate planning aimed at inter alia shortening journey times, ensuring access to water and food and controlling the stocking density in the vehicle.

International journeys, often with varying climatic conditions, place additional demands on the organiser. Longer journeys require even more careful planning, consideration of the veterinary and sanitary–epidemiological requirements of individual countries, and the full documentation necessary for transport between borders. In such situations, it is particularly important to reduce stress on the animals; this can be achieved by driving calmly, minimising noise, controlling temperature, and ensuring a comfortable space in the vehicle. Similar considerations should be made when transporting alpacas to exhibitions.

It follows from the above that, both in national and international transports and at exhibitions, it is very important to follow welfare rules, to prepare transports properly, to constantly monitor health, and to minimise stress. By adhering to these principles and to the regulations in force, it is possible to protect the health and comfort of alpacas, even under demanding conditions.

In South America, alpacas are used for meat production. Many experience inadequate handling prior to slaughter, including during loading, transport, and unloading. This treatment exposes the animals to stress and pain, resulting in increased levels of stress indicators such as cortisol and glucose in the blood; it also increases the risk of injury and bruising. These factors not only impair meat quality (including a change in pH and carcass colour) but also serve as a clear indicator of reduced animal welfare, associated with physical and psychological suffering and weight loss [115,116,117,118,119].

In response, strategies have been put in place to improve the welfare of alpacas, as proper treatment during pre-slaughter is linked to improved meat quality and quantity. The strategies include providing training for the personnel involved in transporting and handling the animals, and upgrading slaughterhouse and transport infrastructure to reduce stress and risk of injury [117].

### 11.2. Alpaca Exhibitions

As alpaca exhibitions play an important role in the assessment of breeding value and selection of genetic material, their organisation requires a high level of animal welfare, especially during transport, quarantine, and presentation. Transport takes place in special boxes with openwork walls, often in air-conditioned aircraft, and continuous veterinary care is provided to minimise stress and protect the health of the animals [120]. Quarantine is an essential part of bio-assurance for export and import; it can last from 40 days, i.e., between Peru and Switzerland, to 90 days, between Peru and Australia, and up to nine months in some countries such as the USA and Canada [120]. Although this procedure protects the health of the animals, its length and associated isolation can have a negative effect on animal welfare. As such, it is crucial to ensure adequate housing and veterinary care during this time.

## 12. Enriching the Living Environment of Alpacas

When enriching the living environment of an alpaca, the approach should be based on an understanding of its natural behaviour. Alpacas actively maintain their condition and well-being through a variety of grooming behaviours. They are often observed scratching and rubbing against rough surfaces such as poles, fences, and walls. They also regularly roll in the sand, as a form of dust bathing [27,28]. The frequency and patterns of these activities throughout the day can serve as indicators of their overall well-being [12]. Interestingly, the quality of well-being is significantly influenced not so much by the size of the enclosure itself, but by the quality of the environment. The presence of enriching elements, such as hills and trees, promotes various beneficial activities, including natural forms of grooming, as does access to varied, low-calorie food and the opportunity to explore the surroundings; alpacas also thrive in the company of their own species and should certainly not be kept in complete isolation, although they can coexist with other farm animals, particularly camelids. In practice, mixed herds work well and pose no problem in daily care. Alpacas are active for most of the day, and a stimulating environment encourages them to interact and be more active, thus promoting greater well-being [17]. Effective forms of enrichment can be physical (poles, hills, varied terrain), nutritional (hay and treats placed in different locations, hidden treats), social (forming groups of alpacas or mixed herds), and cognitive/behavioural (clicker training, mazes, tunnel, new or moving objects). Their introduction influences specific behaviours; they can increase the frequency of scratching and rolling, space exploration and social activity, improve curiosity and increase animal cooperativeness, and help reduce stress and monotony (Table 5).

The living environment of an alpaca can be enriched in many ways that promote well-being and encourage natural behaviour. For example, branches can be provided for browsing, which satisfies their need to chew and explore, and rotating grazing between different areas prevents monotony. Hay should also be placed in various outdoor areas, weather permitting, to encourage the animals to actively forage [17].

Providing smaller portions of hay more frequently, together with varying the locations and methods of feeding, and the use of different seeds and treats, stimulates curiosity and encourages exploration. Elements such as scratching posts and wall-mounted brushes support grooming behaviour, while mirrors can provide visual stimulation. In summer, water features such as sprinklers and shallow wading pools should be installed to aid thermoregulation and promote activity. In practice, introducing different forms of enrichment at regular intervals, such as daily or several times a week, has been found to encourage natural and positive behaviour in alpacas.

When planning pastures, efforts should be made to ensure that the animals have access to outdoor stimuli. The terrain should be varied (e.g., small undulations such as king of the hills) to allow for observation of the surroundings and increase the attractiveness of the space; additional environmental stimulation can be provided by busy areas such as roads, bike paths, and pedestrian crossings [17].

Enriching the environment can entail a variety of sensory-stimulating activities, exploratory behaviours, and cooperation with their caregiver. For example, treat panels or doors with hidden carrots can be provided for the animals to study and obtain treats. Shaking bowls and rotating containers (e.g., plastic jars with holes) with hidden food also engage the alpaca in foraging. In winter, food can be hidden in the snow, which encourages movement. Clicker training, based on positive reinforcement, increases cooperation and facilitates relationship building. It is also worth introducing trust exercises, off-leash play, and encouraging the alpaca to navigate mazes, tunnels, and hoops [17].

Kapustka and Budzyńska [55] found novel objects to evoke either fear, curiosity, or indifference in alpacas. Fear manifested itself through escape and vigilance, curiosity through exploration, and indifference through chewing food or lying down; interest also manifested as more frequent and faster contact with new objects, indicating higher behavioural scores. The response also depended on the type of object: moving and sudden objects evoked stronger fear.

Hence, by introducing a variety of environmental enrichment stimuli, it is possible to support positive behaviour and animal welfare, while tailoring interventions to individual responses.

## 13. The Problem of Unwanted Alpacas

In North America, raising alpacas was heavily promoted as a great investment, especially among retirees, with the promise of high financial returns and easy herd management. In practice, however, plummeting sale prices and growing overproduction have resulted in large numbers of unwanted animals; this was particularly apparent when potential investors purchased ‘3-in-1’ packages, i.e., those including a pregnant female and young cria. The demand was stimulated by a combination of rapid market growth and intensive marketing, in which the alpaca was treated more as a cute ornament or investment than a living creature requiring daily care. However, breeding proved costly and demanding, and falling prices and financial difficulties led to alpacas being abandoned, sold at undervalued prices, or sent to slaughter.

Such activities directly threaten animal welfare, leading to starvation, neglect, stress, disease, and sometimes even death. In response, herd evacuation, veterinary care, and male castration services have been provided by rescue organisations such as Southeast Llama Rescue, Cross Creek Alpaca Rescue in the USA, and the UK’s Alpacaly Ever After CIC; these also prepare the animals for adoption. Due to these initiatives, alpacas are regaining safety, stability, and dignified living conditions. Nevertheless, adoption often remains a challenge due to their lack of adaptation to life on hobby farms, lack of training, tougher nature, and specific care requirements, which can place additional strain on rescue organisations.

Hence, as a result of the surge in demand, the United States is faced with increasing numbers of unwanted alpacas (USAHA, 2015). Most end up in shelters, or are sold over the internet, diverted to auctions, or slaughtered, often with inadequate supervision. Hence, the decision to raise alpacas as a ‘great investment’ actually represented a largely unconscious risk to animal welfare. Examples such as the crisis at Jocelyn’s Alpaca Ranch in Oregon, where more than 170 starving alpacas required immediate assistance, highlight the need for breeder education, proper care, and support for rescue systems [121,122].

## 14. Summary

The well-being of alpacas (*Vicugna pacos*) depends on various interrelated factors, including environmental conditions, nutrition, social relationships, and interactions with humans. In Europe, the optimal housing system combines access to pasture with buildings to provide shelter. It is also particularly important to provide appropriate microclimatic conditions, such as good ventilation, access to daylight, soft ground, and protection from extreme temperatures, and to adapt the space to the needs of different groups, i.e., pregnant females, young animals, or males.

Another important role is also played by human–animal relationships. Early, appropriate socialisation increases trust and facilitates handling; however, care must be taken as excessive physical contact, especially with young males, can result in serious behavioural disorders such as berserk syndrome. Positive training methods, such as Camelidynamics, can be used to reduce stress and improve human–animal relationships [31,37].

Alpacas live in herd structures with a distinct social hierarchy. Conflicts, especially between males, can be intense and dangerous, often requiring separation [46]. Also, the reproductive process should be allowed to proceed in a manner consistent with the natural rhythms of the alpaca and which respects its needs.

Grooming procedures, such as shearing, nail trimming, and dental trimming, are essential for health and comfort; however, they should be performed using stress-reducing techniques, such as gradual familiarisation with touch and gentle restraint. It is recommended that shearing should be performed while standing to reduce stress and the risk of injury [13,14,32,102,103].

Environmental enrichment is crucial for reducing stress and promoting natural behaviours. Stimulating elements such as scratching posts, toys with hidden food, and providing varied terrain promote exploration and activity. Alpacas should not be isolated, as contact with other members of their species is a fundamental social need [18]. In addition, the reaction is also determined by the type of stimulus; moving objects typically trigger more anxiety than static ones [55].

It is important for the handler to monitor the well-being of alpacas regularly. Signs of stress include spitting, freezing, and excessive vocalisation. Signs of well-being include a relaxed posture, natural chewing, and active exploration of the environment [10,11,14,31,37].

Therefore, alpaca handlers should provide their animals with appropriate environmental conditions, balanced nutrition, opportunities to express their natural behaviours, and stress-free management based on positive interactions with humans. They should also monitor their well-being through behavioural observation and adapt breeding practices to current standards.

## Figures and Tables

**Table 1:** Alpaca welfare in the light of the five freedoms—guidelines and risks.

Freedom	Requirements and Recommendations	Indicators/Problem	References
1. Freedom from physical and thermal discomfort	Adequate shelter: buildings with good ventilation or shelters to protect from rain, wind, sun. Suitable substrate: a layer of sand covered with straw (dry, soft, insulating). Avoid bare concrete, i.e., without mulch. Adequate space: min. 2 m^2^ per animal; provide separate pens for different groups (pregnant, juveniles, males) to reduce stress and chance of conflict. Protection against overheating: provision of shade, constant access to water; use of “thermal windows” (thinner skin on abdomen, groin). Preservation of natural “latrines” as an indicator of well-being.	Overheating in summer (alpine animals). Overcooling when kept on concrete without bedding, especially in winter. Improper space management can cause herd conflicts, leading to stress and injuries. Lack of shelter in the paddock can lead to thermal discomfort.	[18,19,20,21,22]
2. Freedom from hunger, thirst and malnutrition	Base the diet on roughage (grass, hay; 80–90% of the diet). Moderate use of concentrated feed; pellets with low starch content are recommended. Constant access to clean, fresh water from open tanks or float-operated drinkers: requirement up to 6 L/day for lactating females. Supplementation with vitamin D (year-round for youngsters, winter for adults) and selenium in regions with deficiencies. Organisation of the pasture: rotational grazing, appropriate stocking density (max 15 alpacas/ha), and providing hay in feeders next to the depleted pasture.	Gastrointestinal tract not adapted to large amounts of sugars and starches → digestive disorders. Long-term use of tissue reserves without replenishment → serious health problems, death. Herd hierarchy → lower individuals may be prevented from accessing food and water, leading to stress and reduced fitness. Alpacas rarely use salt licks → risk of mineral deficiencies.	[12,18,19,23,24]
3. Freedom to express natural behaviour	Herd life: avoid isolation—alpacas are herd animals. Enrichment of the environment: provide hills, trees, scratching posts, games with hidden food, rotational grazing, spreading hay in different places. Provide opportunities for exploration, play (bouncing, chasing in youngsters), and grooming behaviour (rolling in the sand, rubbing). Respect social and sexual behaviour consistent with the herd hierarchy. Maintain circadian rhythms (foraging, resting).	Stimulus-poor environment → frustration, stereotypical behaviour, negative psychological impact. Isolation: severe stress (increased mobility, anxiety, accelerated heart rate). Excessive socialisation with humans (especially in young males) → “Berserk” syndrome (aggression, sexual behaviour towards humans). Inability to create “latrines” → disruption of natural behaviour.	[17,21,22,25,26,27,28]
4. Freedom from pain, injury and disease	Regular grooming: shearing (prevents overheating), nail trimming (prevents lameness, inflammation), tooth correction (prevents difficulty in taking food). Use stress-free grooming methods: immobilisation in a standing position at the shearing (less stress); gentle, gradual habituation to the treatments. Separate males (especially breeding males): avoid fighting and serious injury. Removal of tusks (‘fighting teeth’) in males: reduces the risk of wounds during fighting.	During treatments: spitting, biting, kicking, freezing, vocalising (signs of stress and discomfort). Neglected nails: deformities, lameness, weight loss. Uncorrected teeth: damage to the oral mucosa, ulceration, and eating problems. Male fighting: biting, knocking down other males, and serious injuries to neck, limbs, genitalia.	[11,13,14,15,16,29,30,31,32,33,34]
5. Freedom from fear and stress	Positive relationships with humans: provide early, controlled socialisation; avoid excessive anthropomorphisation and physical contact with very young individuals. Stress-free training: use positive reinforcement methods (e.g., Camelidynamics, clicker training) instead of coercion. Stress-free handling: employ gentle restraint techniques, design pens to facilitate calm herd movement. Minimising stress during treatments: shear gradually, avoid loud noises (e.g., clippers). Welfare monitoring: observation of behaviour (behavioural tests, escape distance), measurement of cortisol in saliva/coat (not in blood).	Signs of fear/stress: spitting, freezing, nervous shifting, tail lifting, loud vocalisation (‘whistles’, growling), hyper-vigilance. Stress during procedures and contact with strangers. “Berserk” syndrome: serious behavioural problems caused by dysfunctional relationships with humans, leading to aggression. Herd conflicts and inability to escape in a confined space → chronic stress.	[3,11,14,25,31,35,36,37,38,39]

**Table 2:** The most common congenital and genetic defects in alpacas and their consequences.

System/Organ	Defect/Irregularity	Health and Husbandry Implications	References
Head and face/Skull	Posterior nostril atresia (choanal atresia), maxillofacial disgenesis (wry face), ear and eye deformation, juvenile cataracts, congenital deafness, blue eye defect	Breathing difficulties, aesthetic and functional impairment	[80,81,82,84,85]
Dentition	Brachygnatia superior or inferior	Abnormal occlusion, difficulty in taking food	[83]
Musculoskeletal system/limbs	Arthrogryposis (joint stiffness), joint deformities, syndactyly, polydactyly, ankle bone rotation	Limited mobility, problems in breeding, hereditary risk of transmission of defects	[80]
Heart	Ventricular septum defects	Restriction of heart function, potentially life-threatening	[80,84]
Reproductive system—females	Ovarian hypoplasia/aplasia, uterine and vaginal aplasia, uterus unicornis, cervix duplex, clitoral hyperplasia	Infertility, intersex	[80,85,86,87]
Reproductive system—males	Testicular hypoplasia, cryptorchidism, hypospadias, persistent phrenulum, ectopic testes, ‘corkscrew’ penis	Effects on fertility; incidence of testicular hypoplasia	[80,85,86]
Chromosomal and genetic aberrations	Monosomy X, trisomy X, XX/XY chimerism, autosomal translocations	Intersexism, pseudohermaphroditism, infertility	[80,88,89,90]
Other/various	Atresia ani, atresia coli, gastrointestinal malformations	Congenital, sometimes associated with recessive mutations	[80]

**Table 3:** Most common limb defects and deformities in alpacas (*Vicugna pacos*) and their impact on health and welfare.

Defect/Irregularity	Health and Welfare Implications	References
Angular limb deformities (valgity of the wrists, knees)	Difficulty walking, joint pain, reduced mobility, risk of injury and secondary arthritis	[91,92]
Syndactylia (knit fingers)	Mobility difficulties, risk of injury, reduced limb function	[93]
Polidaktylia (additional fingers/toes)	Walking disorders, risk of injury, balance problems	[93]
Arthrogryposis (stiffness of limb joints)	Restriction of mobility, joint pain, difficulty walking and taking food	[80]
Bone mineralisation defects (rickets, osteomalacia)	Limb deformities, pain, reduced mobility, risk of fractures	[93]
Limb deformities related to trauma or poor footing	Pain, restricted mobility, chronic degenerative joint changes	[94]

**Table 4:** Indicators and methods for assessing alpaca welfare according to the Five Domain Model.

Domain	Behavioural/Physiological Indicators	Measurement Methods	Interpretation	Implications for Management	Authors
Nutrition	Physiological: BCS (Body Condition Score), micronutrient levels (vit. D, selenium), digestive parameters Behavioural: mastication and rumination, hierarchy at feeders, time spent foraging	Observation of BCS, blood analysis (micronutrients), observation of feeding behaviour (ethogram, video recordings), monitoring of feed and water intake	Normal BCS (3 points) Normal blood levels of vit. D (at least 50 nmol/L) and selenium (0.5–0.7 µmol/L) Balanced foraging and rumination times (foraging 5–6 h per day, ruminating food 8–9 h)	Diet: 80–90% hay/grass Restriction of starchy concentrates Vitamin D and selenium supplementation according to age and region Multiple feeding and watering points	[18,19,24,43,44,45,95,96,97]
Environment	Physiological: body temperature, microclimate parameters Behavioural: use of shade, water, rolling, use of latrines, reactions to danger	Infrared thermography, microclimate measurement, behavioural observation (ethogram, video recordings), escape distance measurement	Normal body temperature (35.1 to 39.4 °C) Escape distance close to zero (tame) Presence of natural behaviour	Shelter + pasture Soft ground (straw on sand) Permanent access to shade and water in summer Minimum area of 2 m^2^/pcm Solid pasture fencing	[18,19,20,21,22,30,38,46,52,98,99]
Health	Physiological: FGM (faecal glucocorticoid metabolites), cortisol in saliva, HRV (heart rate variability), nail length and occlusion Behavioural: activity level, social interactions, body posture	FGM measurement (prolonged stress), salivary cortisol concentration (acute stress), HRV, clinical examination, observation	Normal FGM (12 to 47 ng/g faeces) Normal salivary cortisol (0.6 to 3.5 nmol/L), Higher heart rate variability (HRV, e.g., SDNN) is more favourable Correct nail length and bite	Regular correction of nails and teeth Gentle immobilisation methods Monitoring herd health	[13,14,15,16,32,33,34,99,100,101,102,103,104]
Behaviour	Behavioural: positive submissive signals, grunts, feeding together, youngsters playing, Flehmen response, “orgling”, proximity to the mother; negative aggression, alarm reactions, isolation, sucking patterns	Herd observation (etho-gram, video recordings, live observation), re-registration of behavioural frequencies, separation and re-introduction tests, monitoring suckling, cortisol in saliva/coat	Minimal aggression Strong social ties Presence of natural behaviour Proper suckling and development of young	Avoid mixing non-breeding males Castrate non-breeding males Provide space to avoid conflicts Minimise interference at birth and weaning	[9,15,26,27,46,56,57,62]
Emotional/mental state	Behavioural: positive approach to the carer, acceptance of touch, walking on a loose rope, relaxed posture; negative avoidance, escape, kicking, freezing Physiological: Cortisol in saliva during treatments	Measurement of escape distance, human interaction tests, observation of cooperation, analysis of socialisation histories, measurement of cortisol before, during and after treatment	Zero escape distance to a known caregiver Eager and calm cooperation Low cortisol with low-stress methods	Positive reinforcement (clicker, Camelidynamics) Controlled early socialisation Staff training in gentle handling methods Preferred shearing in standing position Gradual familiarisation with equipment Regular observation and interaction	[15,16,19,38,46,52]

**Table 5:** Types of environmental enrichment for alpacas and their impact on behaviour and welfare.

Form of Enrichment	Example	Effect on Behaviour and Welfare	Frequency of Behaviour
Physical/structural	Posts, fences, scratching brushes; hills, trees, varied terrain; sprinklers, shallow paddling pools	Increased scratching, rolling, exploration; improved thermoregulation and physical condition	Scratching and rolling several times a day; regular climbing and exploring throughout the day
Nutrition/dietary	Hay and treats placed in various locations; hidden treats in panels or snow; rotational grazing	Stimulated curiosity and exploratory activity; reduced monotony and stereotypes	Seeking food several times a day; more activity when searching for hidden food
Social	Homogeneous groups of alpacas; mixed herds with other camelids	Increased social and nurturing behaviour; reduced isolation stress	Social interaction almost throughout the day; mutual cleaning and physical contact
Cognitive/behavioural	Clicker training, confidence exercises; tunnels, mazes, hoops; moving or new objects	Improved cognitive abilities and curiosity; increased cooperation with the animal; observation of individual reactions (fear, curiosity, indifference)	Interaction and exercise daily or several times a week; reactions vary according to type of object

## Data Availability

Not applicable.
